# Self-assembling, protein-based intracellular bacterial organelles: emerging vehicles for encapsulating, targeting and delivering therapeutical cargoes

**DOI:** 10.1186/1475-2859-10-92

**Published:** 2011-11-03

**Authors:** José L Corchero, Juan Cedano

**Affiliations:** 1 CIBER de Bioingeniería, Biomateriales y Nanomedicina (CIBER-BBN), Barcelona, Spain; 2 Institute for Biotechnology and Biomedicine, Universitat Autònoma de Barcelona, Bellaterra, 08193 Barcelona, Spain; 3 Laboratory of Inmunology, Regional Norte de la Universidad de la República, 50.000 Salto, Uruguay; 4 Department of Genetics and Microbiology, Universitat Autònoma de Barcelona, Bellaterra, 08193 Barcelona, Spain

**Keywords:** self-assembling proteins, protein delivery, nanoparticles

## Abstract

Many bacterial species contain intracellular nano- and micro-compartments consisting of self-assembling proteins that form protein-only shells. These structures are built up by combinations of a reduced number of repeated elements, from 60 repeated copies of one unique structural element self-assembled in encapsulins of 24 nm to 10,000-20,000 copies of a few protein species assembled in a organelle of around 100-150 nm in cross-section. However, this apparent simplicity does not correspond to the structural and functional sophistication of some of these organelles. They package, by not yet definitely solved mechanisms, one or more enzymes involved in specific metabolic pathways, confining such reactions and sequestering or increasing the inner concentration of unstable, toxics or volatile intermediate metabolites. From a biotechnological point of view, we can use the self assembling properties of these particles for directing shell assembling and enzyme packaging, mimicking nature to design new applications in biotechnology. Upon appropriate engineering of the building blocks, they could act as a new family of self-assembled, protein-based vehicles in Nanomedicine to encapsulate, target and deliver therapeutic cargoes to specific cell types and/or tissues. This would provide a new, intriguing platform of microbial origin for drug delivery.

## Introduction

Our view of the intracellular bacterial cell organization has significantly changed over the past years. Once taken simply as reaction vessels containing a homogeneous solution of proteins, bacteria are now seen as organisms with an intricate subcellular architecture in which individual proteins localize to particular sites within the cell, often in a dynamic manner [1-4]. Protein-based microcompartments are particularly intriguing intracellular bacterial organelles. They are large macromolecular complexes consisting of metabolic enzymes encapsulated within multiprotein, polyhedral shells, reminiscent of the viral capsids structures. A common feature of such bacterial microcompartments (BMC) is a thin shell primarily (composed by a few thousand protein subunits, so-called BMC shell proteins) that encapsulate the enzymes while allowing transport of substrates and products. Several studies indicate that the general role of the protein shell is to seclude toxic or volatile metabolic intermediates, while allowing enzyme substrates, products and cofactors to pass. Polyhedral organelles had been identified and visualized by electron microscopy in cyanobacteria and some chemoautotrophs, and were first isolated in 1973 and determined to contain the CO_2_-fixing enzyme RuBisCO [5,6]. They were named carboxysomes, and are now recognized as the first member of a diverse group of microcompartments. BMC proteins were later found to be encoded in the propanediol utilization operon (*pdu *operon) of the heterotroph *Salmonella *[7] and by an operon for metabolizing ethanolamine (*eut *operon) in enteric bacterial species, including *Salmonella *and *Escherichia *[8]. Until recently, the diversity of these structures had been overlooked because many of them are not formed on standard growth media and because their observation requires the use of electronic microscopy techniques. However, recent genome analyses have detected seven functionally distinct organelles distributed among over at least 40 bacteria genera. Searches for homologies with shell proteins in protein sequence databases have emphasized the widespread occurrence of microcompartments across bacteria species, and the likely horizontal gene transfer for their genetic determinants spread [9].

### Main polyhedral organelles in bacteria

#### Carboxysomes

Isolated in the early 1970s, carboxysomes were the first bacterial polyhedral organelle identified [5,6]. They are polyhedral inclusions of approximately 100-150 nm in cross section, and with a 3-4 nm protein shell composed of six to ten different protein species. These organelles have been identified in cyanobacteria and in many chemoautotrophic bacteria, but not in eukaryotes [10-12]. Their distribution and composition has been investigated by genomic and phylogenetic analyses [13]. Their structure varies among different producing bacteria, and according to their constituent proteins, they can be divided into subtypes α and β. The α-type carboxysome is that of the facultative chemoautotroph *Halothiobacillus neapolitanus*. Type β carboxysomes are found in the β subdivision of the cyanobacteria, where carboxysome proteins are designated CCM because of their role in a carbon dioxide concentrating mechanism. The function of carboxysomes is to enhance autotrophic CO_2 _fixation at low CO_2 _levels. This role is supported by the findings that carboxysome formation is induced by CO_2 _limitation [14], and that mutant strains of cyanobacteria and chemoautotrophs unable to properly form carboxysomes require high CO_2 _levels for autotrophic growth [15,16]. The carboxysome is filled with the ribulose bisphosphate carboxylase/oxygenase (RuBisCO) enzyme [6], which catalyzes the CO_2 _fixation step in the Calvin cycle. Also associated with the carboxysome is the carbonic anhydrase (CA) enzyme, responsible of the conversion of HCO^-^_3 _(that is not used by RuBisCO) to CO_2_, the substrate for RuBisCO [17-19].

### Organelles for 1,2-propanediol utilization (*pdu*)

Carboxysomes were the only known polyhedral organelles for many years. However, *Salmonella enterica *was found to form a polyhedral organelle during growth on 1,2-propanediol (1,2-PD) as a sole carbon and energy source [20]. Electron microscopy showed that *S. enterica *forms structures (similar in size and shape to carboxysomes) during growth on 1,2-PD but not during growth on other carbon sources [20,21]. Microscopy analysis showed that coenzyme B12-dependent diol dehydratase (DDH) is a major component of the pdu organelles, and that the PduA protein is a shell component [20,22], together with other 14 different polypeptides. Genetic studies showed that genes specifically involved in 1,2-PD utilization encode homologs of the carboxysome shell proteins [7], but that their enzyme cargo and physiological functions are clearly different [21-23]. The pdu organelles function is to minimize the harmful effects of a toxic intermediate of 1,2-PD degradation (propionaldehyde) [21-23]. Mutants unable to form pdu organelles undergo a 20-h period of growth arrest during degradation of 1,2-PD, whereas wild-type *S. enterica *grows normally under similar conditions [21]. The length and severity of growth arrest increases at higher 1,2-PD concentrations, suggesting that it results from the accumulation of a toxic metabolite derived from 1,2-PD [21].

### Organelles involved in coenzyme B12-dependent ethanolamine degradation (*eut*)

It has also been described a polyhedral organelle involved in ethanolamine utilization (*eut *operon) by *S. enterica *[8]. The Eut and Pdu microcompartments share some homologous enzymes: both metabolic pathways proceed via aldehyde intermediates, propionaldehyde in the case of Pdu and acetaldehyde in the case of Eut [2]. The Eut microcompartment function is to metabolize ethanolamine without allowing the release of acetaldehyde into the cytosol, thus alleviating the potentially toxic effects of excess aldehyde in the bacterial cytosol [24-26] and also preventing volatile acetaldehyde from diffusing across cell membrane and leading to a loss of carbon [27]. The initial step of this process is catalyzed by coenzyme B12-dependent ethanolamine ammonia lyase, which converts ethanolamine to acetaldehyde. Subsequently, acetaldehyde is converted to ethanol and acetate by a series of reactions analogous to those used for 1,2-PD degradation [28]. This suggests that Eut and Pdu organelles might have the common function of protecting cells against aldehyde toxicity [29]. In addition, recent studies (referring to the Eut organelles as metabolosomes) suggest that these structures function concentrating both enzymes and their substrates to allow a more efficient growth while minimizing acetaldehyde toxicity and maintaining coenzyme A balance [30].

### BMC shell structure

**Figure 1:**
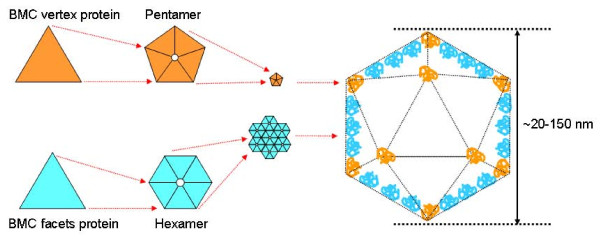
**Model for the hierarchical assembly of a typical bacterial microcompartment**. The facets of these icosahedral nano-cages are made of one type of protein (in light blue) that further self-assemble to give hexamers. On the other hand, the vertices are formed by pentamers resulting of the self-assembly of a different protein (in orange). Pores allowing traficking of molecules are located at the center of each hexamer and pentamer. Sizes can range from 20-25 nm for the encapsulin shell to 100-150 nm for carboxysomes.

At present, about 1700 proteins containing BMC domains have been identified, covering at least 10 different bacterial phyla. Multiple paralogs of the shell proteins are essentially always found together. The typical BMC domain consists of 90 amino acids in length with an alpha/beta fold pattern [9,31]. BMC proteins self-assemble to form disc-shaped hexamers, the basic building blocks of the shell (Figure 1, light blue proteins). Each hexamer typically presents a narrow pore through the middle, along the six-fold axis of symmetry. Crystal structures show that such hexamers further assemble side-by-side, forming a molecular layer [9,32,33], with 4- to 6-Å-wide gaps between hexamers. Both central hexamer pores and the gaps between hexamers potentially serve as gates for metabolites, selectively allowing passage of negatively charged molecules such as the substrates and products of RuBisCO while restricting uncharged molecules such as CO_2 _and O_2 _[9]. A notable feature of the hexagonal layer of the carboxysome shell is the presence of a bowl-shaped depression or concavity on one side of the hexameric building block, side where both N-termini and C-termini are usually located. This molecular layer appears to represent the flat facets of the shell.

Electron microscopy studies have confirmed that carboxysomes are approximately icosahedral in shape. The construction of large icosahedral structures typically requires a combination of hexameric and pentameric units. Pentamers generate curvature in an otherwise flat hexagonal sheet, occupying the vertices of the icosahedral shell. In agreement with this vision, homologous proteins CcmL and CsoS4A from two different types of carboxysomes self-assemble to form pentamers (Figure 1, orange proteins), whose size and shape are compatible with their placement at the vertices of an icosahedral shell containing 12 pentamers [32]. Both CcmL and CsoS4A proteins were expressed in *Escherichia coli *and an atomic model was built and refined at a resolution of 2.4 Å and 2.15 Å for CcmL and CsoS4A, respectively. The study of such crystal structures confirmed that both CcmL and OrfA formed symmetric pentamers [32]. This model of CcmL or CsoS4 pentamers occupying the icosahedral vertices is in agreement with their low abundance in the shell, and with mutational experiments in which deletion of the genes led to failure to form closed shells [34]. However, there is not a total consensus on the role of CcmL or CsoS4 proteins with respect to provide the needed curvature in order to form icosahedral, closed shells. Some studies [35] have shown that CsoS4 proteins are apparently not essential determinants of carboxysome shape: a *Halothiobacillus neapolitanus *knockout mutant that does not produce CsoS4 predominantly forms carboxysomes of normal appearance, in addition to some elongated microcompartments. However, and despite their normal shape, mutant carboxysomes are functionally impaired (in the absence of CsoS4 protein, the carboxysome shell loses its permeability to CO_2_).

This scenario of hexamers forming flat facets and pentamers providing the needed curvature in the overall icosahedral structure (as shown in Figure 1) is not universal for all types of bacterial microcompartments. For example, the homologous protein of CcmL and CsoS4 in the Eut microcompartment is EutN, whose structure is hexameric rather than pentameric. The difference between the oligomeric state of EutN, compared to CcmL and CsoS4, presumably reflects structural differences between the Eut microcompartment and the carboxysome. Another alternative is that the Eut microcompartment could lack pentamers, which would explain why enteric bacteria BMC tend to have less-regular icosahedral shapes than carboxysomes. Purified Pdu microcompartments contain two major BMC-domain proteins (PduA and PduJ) that are closely related in sequence to the hexamers proposed to form the faces of the carboxysome [22]. The *pdu *operon also encodes a homolog (PduN) of the pentamer proposed to from the vertices of the carboxysome [32]. These analogies suggest that Pdu shell may have flat faces formed by PduA and PduJ hexamers, and vertices made from PduN pentamers. However, and as seen for the Eut microcompartment, electron microscopy shows that Pdu microcompartments are more irregular in shape than carboxysomes: Pdu (and Eut) microcompartment does not resemble a regular icosahedron as closely as the carboxysome does, fact that may correlate with the different behaviors of the CcmL/CsoS4/EutN/PduN family of proteins.

### Encapsulating therapeutic proteins into protein-based bacterial organelles

As the number of therapeutics requiring parenteral administration increases, delivery vehicles improving therapeutic efficacy will find increasing use [36-39]. Among all the currently available vehicles, the utility of nanoparticles to deliver chemotherapeutics *in vivo *is now well established, with several formulations and synthesis protocols available [40-42]. They can be synthesized from a range of materials, being the most common ones based on biocompatible, and preferably biodegradable, lipids or polymers [40]. In general, nanoparticles are designed to minimize limitations of conventional drug delivery systems, including nonspecific biodistribution and targeting, low aqueous solubility, poor bioavailability and low therapeutic effects due to insufficient drug concentration at disease sites. Moreover, nanoparticles of submicrometer size offers clear advantages over microparticles as they are best suited for intravenous delivery [43].

Macromolecular self-assembly has been exploited recently to engineer materials for the encapsulation and controlled delivery of therapeutics. Self-assembled structures can be formed by a variety of building blocks, both organic and inorganic [44]. Peptides and proteins are among the most useful organic building blocks, since they are stable and robust and can spontaneously associate to form nanotubes, nanospheres, nanofibrils, and other ordered nano-sized structures [45,46]. For example, since it was described that protein aggregation as bacterial inclusion bodies does not necessarily mean the inactivation of the forming proteins [47], such structures incorporated to the list of self-assembling materials with putative biotechnological and biomedical applications [48]. When protein self-assembling does not occur spontaneously, some strategies to promote it can be used. For example, fusion of self-assembling or arginine-rich peptides has been reported to promote the formation of active protein aggregates in *E. coli *[49,50] or of structured nanodisks [51], respectively.

Protein-based capsids (also known as protein nano-cages or nano-containers) are appealing vehicles for drug delivery [52,53]. Their biocompatibility, biological fabrication, functional diversity and versatility of design though protein engineering (assisted by in silico instruments) make them extremely powerful materials. A well-studied example of natural protein nano-cage is ferritin, a 450 kDa protein assembly of 24 subunits. Ferritin, ubiquitous in cells and in extracellular matrices, is a cage-like protein with an inner space of 8 nm in diameter. This inner space allows storage of up to 4500 Fe atoms as ferric oxyhydroxide clusters, serving as a reservoir providing Fe atoms for metabolic use [54]. Viral capsids, and other protein cages, are increasingly being used as multivalent, multifunctional nano-containers. Mimicking viral structures as models, several protein-only nano-cages have been explored for materials applications [55].

**Figure 2:**
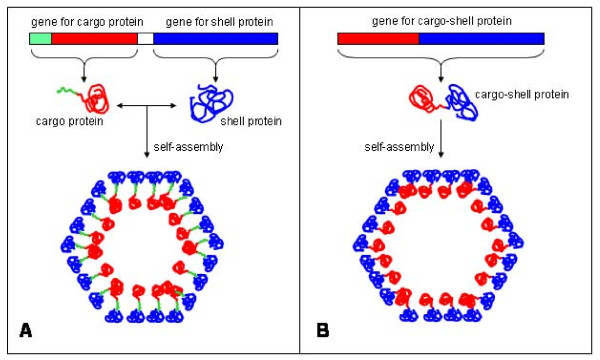
**Targeting and encapsulation of proteins into BMCs nano-cages**. In some cases (panel A), a stretch of ~15-20 aminoacids (in green) located at the N-terminus of the inner cargo protein (in red) directs and binds it to specific sites on the inner surface of the shell protein (in blue). For other bacterial microcompartments (panel B), the cargo protein (in red) is synthesized together with the shell-forming domain (in blue) from one unique gene.

Mechanisms directing enzyme encapsulation within protein-based bacterial organelles have been studied and revealed during the last years. In some cases (Figure 2, pannel A), a stretch of a few (~15-20) aminoacids at the N-terminus of the inner cargo protein directs and binds it to specific sites on the inner surface of the shell protein. When such directing peptide is not present, the strategy is to synthesize the cargo protein together with the shell-forming domain from one unique gene (Figure 2, pannel B).

As an example of the first strategy (shown in Figure 2, pannel A), in β carboxysomes the protein CcmM is used as a scaffold to form interactions between both shell proteins and enzymes [56,57], through a CcmM C-terminal region with homology to the small subunit of RuBisCO [58], and probably mimicking the native interactions of small and large subunits of RuBisCO inside the carboxysome. In Pdu microcompartments some of the internal enzymes are also directed to the interior by special N-terminal targeting sequences [34,59]. Fan and colleagues [59] demonstrated that a short N-terminal peptide is necessary and sufficient for packaging enzymes into the Pdu microcompartment. Deletion of 10 or 14 amino acids from the N-terminus of the PduP enzyme (normally found within this microcompartment), significantly impaired its packaging, without affecting its enzymatic activity. On the other hand, fusion of the 18 N-terminal amino acids from PduP to GFP, GST, or maltose-binding protein resulted in the encapsulation of these proteins within the Pdu microcompartment. It is worthy to note that the Pdu shell can be assembled heterologously in the absence of native interior enzymes [34], and that carboxysomes can self-assemble *in vivo *when RuBisCO has been deleted [60], offering appealing opportunities to manipulate such nano-cages in the laboratory in order to fill them with therapeutic molecules. Also in this line, another landmark study about enzyme encapsulation was provided by Sutter and colleagues [61]. They described the smallest (20-24 nm of diameter) known protein-based organelle (found in the hyperthermophilic bacterium *Thermotoga maritima*). The protein family was initially named linocin [62], but it was renamed by the authors as "encapsulins". In many bacteria, the encapsulin gene is positioned downstream within an apparent two-gene operon, being preceded by the gene for either an iron-dependent peroxidase (DyP) or a protein closely related to the iron transporter ferritin (Flp). Sequence alignment of DyP and Flp genes revealed that only those followed by the encapsulin gene carry a C-terminal extension with a conserved amino acids sequence responsible for the protein's physical interaction with the encapsulin protein, by binding to distinct pockets on the interior of the nanocompartment surface.

Support for the existence of the second strategy (Figure 2, pannel B) to encapsulate cargo proteins is provided by the hyperthermophilic archaeon *Pyrococcus furiosus*, where a Flp coding sequence (without any targeting sequence directing its encapsulation by physical interaction with BMC proteins) is fused in frame with an encapsulin gene [61]. In this situation, cargo and encapsulin proteins are synthesized as a fusion that further self-assembles to form a nano-cage containing the cargo protein.

Specific targeting sequences could be of use in biotechnological applications to package proteins inside the stable self-assembled icosahedral shell of encapsulin. As an example of the utility of such approach, an icosahedral enzyme complex, lumazine synthase, was engineered to encapsulate target molecules by means of charge complementarity. The lumazine synthase from *Aquifex aeolicus *(AaLS) represents the container component, as it forms icosahedral capsids large enough to encapsulate proteins. To engineer the charge environment within the capsid, four residues of each monomer that project into the lumen (Arg83, Thr86, Thr120, and Gln123) were mutated to glutamates, introducing extra negative charges to the inner surface of the protein cage. The hypothesis of the authors was that adding a short stretch of positively charged amino acids to a cargo protein should promote its specific encapsulation within this modified proteinaceous nano-container, as was demonstrated by using a modified GFP containing ten arginine residues [63].

### Use of protein-based bacterial organelles as drug delivery system

Once the BMC is loaded with a therapeutic cargo protein, its administration would depend on the target to reach, adopting for each case the most appropriate way. A parenteral administration should be the most common choice to administrate this therapeutic agent, but not the only one. For mouth or throat cancer, oral administration would be a more appropriate method. On the other hand, to treat myeloblastomas the way would be the cerebrospinal fluid.

The field of cell-specific targeting has been significantly advanced, identifying targeting peptides that target their cargos to the vasculature of a variety of tissues, organs, and tumors [64,65]. It would be possible to add on the surface of BMC nanocages any type of specific binding domain, including targeting peptides or antibodies, providing the desired specificity. In this line, it was shown [66] that genetic addition of a targeting peptide (RGD-4C) or chemical conjugation of an anti-CD4 monoclonal antibody onto the surface of the small Hsp cage structure conferred specific cell targeting capacity. In addition, authors were able to load a cargo molecule, a fluorescent imaging agent (fluorescein), within the interior cavity of the Hsp cage, demonstrating the multifunctional capacity of protein nanocage architectures and their potential uses in medicine.

After reaching target cells, release of the cargo is another important issue for drug delivery. One strategy for particle disassembly and drug release is to take advantage of the increase in acidity once these macromolecules are taken up by the endocytotic pathway, where drug release would take place after BMC degradation in the lysosome [67]. In this line, it has been investigated the potential of using histidines for introducing pH-dependent disassembly [68,69]. The results demonstrated that modulating the degree of electrostatic repulsion through changing the number of histidine interactions at subunit interfaces could be a generally applicable strategy for designing pH-triggered assembly/disassembly in protein macromolecular structures.

The use of BMC as a drug delivery system arises some general concerns, like its putative antigenicity. It could be modulated not only by engineering the exposed sequence/s of the constructs, but also by means of post-transcriptional or chemical modification (like pegylation [70]). Other materials used as a carriers (like Teflon, titanium, silicone oil, etc.) are not naturally processed by cells, sometimes remaining forever in the body of the patients and triggering unwanted reactions. On the contrary, due to its proteic nature, recycling by the cells of the BMC carrier would be relatively easy, only requiring the use of one of their multiple proteolytic pathways.

## Conclusions

In protein-based therapies, there are several known issues to be carefully considered, like a short protein half-life *in vivo*, side effects caused by the multiple or high doses that must be administered in order to reach the desirable concentration in the cell, or possible protein denaturation during manipulation. To address such problems, carriers act as a vehicle loaded with high concentrations of therapeutics, providing simultaneously a protective environment, either for local or systemic delivery, thus increasing the drug lifetime. The use of protein cages of non-viral origin as nanomaterials for biomedicine and biotechnology provides a number of unique advantages over other types of vehicles for drug delivery. Their biological origin makes them both amenable to genetic modification and large-scale production. Genetic modification enables the site-specific introduction of chemical and/or structural functionality onto highly symmetric protein cage platforms. By either chemical and/or genetic subunit alterations, it is feasible to simultaneously add new functions to different particle surfaces to direct cage assembly, encapsulation of a synthetic cargo, or targeting to a specific surface or cell. Regions not directly involved in vehicle assembly are generally more suitable to such modifications without losing the desired cage-like architecture. On the other hand, studies like those performed by Sutter and Seebeck pinpoint cargo protein features necessary for its encapsulation, paving the way for future development of protein-based nano-compartments for several nanotechnological applications. The discovery and understanding of signal sequences able to direct enzyme encapsulation into BMC and the underlying mechanisms of such process are key milestones in our understanding of BMC assembly, and leads the way for the development of these bacterial organelles toward biotechnological and biomedical applications. The simplicity of this system makes it attractive for engineering studies aimed at encapsulating distinct enzymes in order to create new vehicles to encapsulate, target and deliver therapeutic cargoes. There is still a long way ahead to explore in this area, but one can envision a great potential for BMC as drug delivery systems. Nowadays, the pharmaceutical industry is having trouble in discovering new drugs to treat a wide variety of diseases. This problem is being a serious limitation in conditions like cancer. Many of the substances that have proven to be highly efficient in removing tumor cells have been lately discarded due to its high toxicity or lack of specificity. The possibility of having a vehicle providing such specificity, by binding to a specific receptor, opens a wide range of possibilities for the rescue of these substances of low specificity.

The use of protein-based architectures is an exciting and fruitful scenario in the design of nano-cages for drug delivery, based on both protein engineering and the use of microbial cell factories. And although the understanding and putative engineering of protein assembling have a long way ahead, novel principles and promising strategies of protein manipulation point out the possibility of the rational construction of nanoscale protein cages as a viable concept.

## Competing interests

The authors declare that they have no competing interests.

## Authors' contributions

JLC and JC have equally contributed to this work. Both authors read and approved the final manuscript.
